# Reasons for inconsistent condom use by young adults in Mahalapye, Botswana

**DOI:** 10.4102/phcfm.v10i1.1492

**Published:** 2018-05-24

**Authors:** Luhaka Kanda, Robert Mash

**Affiliations:** 1 Division of Family Medicine and Primary Care, Stellenbosch University, South Africa

## Abstract

**Background:**

Botswana is one of the countries significantly affected by the HIV and AIDS epidemic. Despite an extensive preventive campaign, the incidence of HIV remains high. Condoms are an important contributor to prevention of new HIV infections, although they are not consistently used by young adults.

**Aim:**

The aim of this study was to explore the reasons why condoms are not consistently used by young adults.

**Setting:**

Mahalapye District Hospital and Airstrip Clinic, Botswana.

**Method:**

This was a phenomenological qualitative study using individual in-depth interviews. Eleven participants were purposively selected, including six males and five females. Data were transcribed and analysed using the framework method.

**Results:**

All participants acknowledged the importance of utilising condoms to prevent unplanned pregnancies and sexually transmitted infections. Reasons not to use condoms were a need to have a child, implied lack of trust or faithfulness, long-term relationships need to please the partner and decreased pleasure. Other contributing factors were lack of knowledge of benefits, less fear of contracting HIV and AIDS as it can now be controlled with medication, influence of tradition, alcohol and drug abuse, peer pressure, power and gender issues and the refusal of the partner. The female condom was largely rejected by young adults in general and by women in particular because of its size and the perception that it is complicated to insert.

**Conclusion:**

The current preventive campaign against HIV and AIDS needs to take cognisance of the factors affecting decisions on the use of condoms by young adults and the obstacles to their use, particularly the new belief that HIV and AIDS is no longer a significant concern.

## Introduction

There are approximately 36.7 million people living with HIV in the world.^1^ Botswana has one of the highest HIV prevalences, estimated at 18.5% for the general population.^2^

Botswana’s prevention of mother-to-child transmission and treatment programme has achieved significant results in preventing new childhood infections and deaths among adults and children. However, the number of new adult infections continues at a high level and more effective prevention efforts are urgently needed.^3^

The motivation to undertake this study came from observing the increasing number of new HIV infections among young adults, the frequency of other sexually transmitted diseases and the number of abortions and unwanted pregnancies at Mahalapye District Hospital, despite the large distribution of free condoms by government and non-governmental organisations. Condoms are an important component in the prevention of HIV infection if used consistently and provide 80% protection against HIV as opposed to not using them at all or inconsistent use.^4^

Use of condoms; however, by sexually active adolescents is low despite awareness of HIV and the need for safe sex. The South African first national youth risk behaviour survey shows that only 40% of male and 31% of female adolescents always use a condom.^5^ In Burkina Faso, Ghana, Malawi and Uganda, only 24% – 51% of adolescents used condoms in their last sexual encounter.^6^

The female condom is the only female-initiated prevention method that is also known to be safe and effective in reducing the risk of pregnancy and the transmission of sexually transmitted infections. Between 50% and 70% of male and female participants find the female condom to be acceptable.^7^ An acceptability study in South Africa found that only 30% of the female participants used the female condom, but of these 86% said they would use it again and 95% said they would recommend it to friends.^8^

In sub-Saharan Africa, negative attitudes towards condom use are often based on socio-cultural factors.^9^ For example, in a study in the North-West province of South Africa, the following reasons for not using condoms were elicited from participants^10^:

- Condoms reduce sexual pleasure and may have physical side effects.
- Belief that condoms cause HIV, could trigger pain in the kidneys or cause worms.
- Lack of information about condoms.
- Social status: you look cheap by using condoms that are free of charge.
- Financial reasons: women fall pregnant in order to qualify for government grants. Men will not pay for sex if you insist on condom usage.
- Distrust in efficacy of condoms.
- Family planning: the desire to have children.
- Cultural reasons such as polygamy.
- Gender-related reasons: in a patriarchal society men decide when and how sexual intercourse takes place.
- Trust: asking for condom usage in a long-term relationship is seen as a lack of trust.

Gender issues are increasingly being recognised as having critical influences on the HIV epidemic in southern Africa.^11^ A study conducted in Botswana and South Africa showed that it is women whose partners are 10 or more years older, women who are abused and those who are economically dependent on their partners who are less likely to suggest condom use.^12^ Alcohol use was also found to be a major barrier to condom use in Botswana.^13^

Prevention programmes should take into consideration the beliefs and norms of the target audience in order to be more effective.^14^ Adapting the SISTA (Sisters Informing Other Sisters about Topics on AIDS) intervention for South African Xhosa women, it was found that behavioural and not only technical interventions have a role to play in reducing the risk of contracting HIV.^15^ In motivating behaviour change a guiding rather than a directing style may be more effective, and health workers may need more effective communication skills when recommending the use of condoms.^16^

This study aimed to explore the reasons why condoms (female and male) were not consistently used by young adults in Mahalapye despite the government’s current campaign on preventing HIV and AIDS.

## Methods

### Study design

The phenomenological qualitative study design aimed to explore young adults’ attitudes, beliefs and behaviours with regard to condom use through in-depth interviews.

### Setting

Mahalapye is a town located in the Central District of Botswana. The town has an estimated 41 000 inhabitants and is situated along the main road between the capital, Gaborone, and the second largest city, Francistown.^17^ In the 2011 census the Central Mahalapye region was reported to have a population of 119 000, with 52% males and 26% aged between 15 and 29 years, a 22% unemployment rate and 82% literacy rate. The HIV and AIDS prevalence in Botswana was estimated at 21.9% in 2016 among adults and adolescents aged 15–49 years.^18^ Mahalapye has one district hospital and four clinics in the public sector as well as a number of private clinics.

In Botswana, the HIV prevention campaign, ‘Wise Up’ was launched in 2011 and utilised a multimedia approach to raise awareness and promote behaviour change among adolescents and youth.^19^ The campaign aimed to increase knowledge of HIV, increase the consistent use of condoms, increase the number of people testing for HIV and reduce the number of people with multiple partners. In 2013 the Botswana Combination Prevention Project was also launched to evaluate whether a combination of proven HIV-prevention measures introduced into a community could significantly reduce HIV incidence.

The package included the promotion of voluntary male circumcision as well as the scale-up of HIV testing, counselling and provision of antiretroviral medication.^20^

### Selection of participants

Purposive sampling was used to select participants from patients presenting with urogenital complaints at Mahalapye District Hospital and Airstrip Clinic. Participants aged between 18 and 28 years, who were sexually active and willing to discuss their use of condoms, were included in the study. An equal number of men and women were selected. Interviews continued until no new themes were obtained from the last two interviews.

### Data collection

Patients meeting the selection criteria were identified in the waiting room by the research assistant, who was a nurse, or by the doctor during the consultation. Patients were then invited to participate and if they consented were interviewed immediately after the consultation or given an appointment to return for the interview at a mutually convenient future date. All the interviews were conducted by the principal researcher between February and December 2015. The interviews were conducted in another more neutral room at the health care facility. The research assistant also translated the conversation during the interviews if necessary because the principal researcher was not fluent in the local language, Setswana.

Data were collected by means of individual in-depth interviews, which were audio-taped. Communication skills such as the use of open-ended questions and active listening were necessary to allow participants to elaborate freely on their ideas. The principal researcher was conscious of his own knowledge and beliefs about the topic and the negative effect he might have as the doctor on participants’ freedom to express their views. He accepted the viewpoints expressed without judgment, utilised active listening skills and did not express his own views. The interview guide was used to prevent missing important points during the interviews. The opening question was, ‘How do you feel about the promotion of condoms to prevent HIV and AIDS?’ Other topics discussed were their reasons not to use condoms, any obstacles experienced in the process of using a condom, their views about the female condom, how practical was it compared with the male one, their critique on the current preventive campaign and any suggestions for improvement. A pilot interview was conducted to check the usefulness of the interview guide and the communication skills of the interviewer. No changes were made to the guide and the interviewer received feedback from their supervisor on the communication skills.

### Data analysis

The audio-taped interviews were transcribed verbatim prior to the analysis. The framework approach was used for data analysis^21^:

- Familiarisation: The transcripts were thoroughly read and the audio-taped interviews listened to and checked against the transcripts for accuracy.
- Thematic indexing: Based on the initial familiarisation with the data, key codes were listed and organised into categories. The construction of the thematic index was validated by both the local family physician and supervisor.
- Coding: All the data were then coded using the thematic index.
- Charting: Data related to each category were then grouped together to form charts.
- Mapping and interpretation: The charts were interpreted and the key themes identified.

Atlas.ti software was used to facilitate the data analysis.^22^ After the interpretation of data, emergent themes were presented to six participants for validation. The supervisor also audited the process of analysis.

### Ethical considerations

After approval by the Health Research Ethics Committee at Stellenbosch University (N10/10/334), the proposal was also approved by the Research Committee of the Ministry of Health (Botswana).

## Results

### Participants’ profiles

Eleven interviews were conducted over a period of 11 months. Although no new themes emerged after the first nine interviews, two more interviews were conducted to ensure saturation of themes was achieved. Participants were mostly in their mid-20s to late 20s; six were males and five were females; most were employed or in higher education (Table 1).

**TABLE 1:** Participants’ profiles.

Participants	Gender	Age (years)	Occupation
P1	Male	26	Assistant pharmacist
P2	Female	28	Nurse
P3	Male	25	College student
P4	Female	23	University student
P5	Female	19	College student
P6	Female	24	Unemployed
P7	Male	25	Sales assistant
P8	Female	24	Sales assistant
P9	Male	25	College student
P10	Male	23	Technician
P11	Male	28	Technician

### Key themes

The key themes are presented below in sections related to personal reasons for using or not using condoms, other obstacles to the use of condoms, attitudes towards the female condom and opinions regarding the government’s HIV prevention campaign.

#### Personal reasons to use or not use condoms

Participants were unanimous on the importance of condoms for the prevention of unplanned pregnancies and sexually transmitted infections, including HIV and AIDS: ‘The use of condoms to me is very safe. The condom is used to prevent the spread of HIV and other STDs and to prevent unwanted pregnancies’ (P4, female, university student).

Participants gave many reasons for not using condoms. Condoms were perceived to be something that was used at the beginning of a relationship when there was still a lack of trust and commitment. Conversely the need to continue using a condom could be seen as a sign of distrust or a lack of real love. Women who wanted to fall pregnant did not use a condom and sometimes falling pregnant was seen as a way of cementing the relationship and ensuring ongoing support. Women also wanted to preserve their relationship by pleasing their partner or making sex more enjoyable by not insisting on a condom:

‘One reason may be where there is trust, meaning having spent some time with the person and that you have tested before (for HIV). You therefore feel secure with that person.’ (P2, female, nurse)

‘You won’t use a condom because you want to have a family.’ (P10, male, technician)

‘That is the mentality of the people of today. They believe sex is the deal. After sex, you can get married or something. It comes with the thought that if you get pregnant, the guy will be so attached to you because of the child.’ (P8, female, sales assistant)

‘Sometimes we, the youth, like to sacrifice. I have been trying to please my man.’ (P4, female, university student)

‘For men, if you ask them the difference when using a condom and not using it, they will tell you of the pleasure of [*sex*] without using condom; they can enjoy it more and more. But when using it, it’s like something else. They don’t really feel that pleasure.’ (P4, female, university student)

#### Major obstacles in the use of condoms

Participants mentioned a number of obstacles in using condoms. One of these obstacles was the attitude of young adults, who think that this is their time to experience life in its fullness without restrictions – to experience different partners and sexual pleasure. Sometimes they also lost control while sexually aroused and neglected to use a condom:

‘I will say the problem with us is that we think in life, there is that stage where we must go crazy and do a lot of things.’ (P1, male, pharmacy assistant)

‘When you are in that moment you don’t think straight. Anything can happen, especially when you are at that stage. If someone tells you there is no condom around, you are not going to pause and go to the shops to look for them. You will just get on with the business.’ (P8, female, sales assistant)

Participants reported that most of the information about HIV and AIDS and condoms was learnt through the media. It was hard to discuss these issues at home with parents and relatives. Conversations about sexual issues were still considered as taboo in many families. There was some confusion on which advice to follow because of the gap between what they learnt from media and at home. Some young adults were just ignorant of the risks of unprotected sex and lacked knowledge of the benefits of using condoms:

‘The other reason why we youth do not use protection is that our parents do not talk of this at all times. I grew up with my family, my mother and father. We were never taught about how to be safe, how to practise those things.’ (P7, male, sales assistant)

‘It’s about some people are maybe not aware of the advantages of condom use. It might therefore be difficult for those ones to use them. There are misconceptions.’ (P5, female, college student)

Gender inequality also played a role. Generally the male partner was the one who decided on the use of a condom and if he was not willing, then it was most likely not used. Participants reported that alcohol and illicit drug use also made youth vulnerable and less likely to use condoms. If one of the partners was drunk, the condom was either not correctly used or not used at all. Women were less empowered financially compared with men and might see their relationship as a way of obtaining money and gifts, sometimes to the extent of dating much older and richer men. This situation made them less likely to negotiate the use of condoms.

There was also a problem with the distribution of free condoms. Usually they were openly displayed in public places like hospitals and clinics. Young adults found it difficult to pick up these condoms in front of everybody for fear of stigmatisation. Likewise, buying condoms in the shops was also challenging for them. Furthermore, the freely distributed condoms were not accepted by all women because of the smell and the reported rash they cause:

‘Women depend on men. If he wears a condom, they don’t have any problem. If he doesn’t, they don’t have a problem either.’ (P1, male, pharmacy assistant)

‘It’s very hard for them to resist to that because they look up to those luxurious stuffs like cell phones, money and all that. Therefore, it is very hard for them to resist doing unprotected sex when they do not want to.’ (P7, male, sales assistant)

‘Yes, alcohol abuse and drugs can lead you to doing sex without condoms because you will be drunk.’ (P4, female, university student)

‘I would not personally get into a store and buy a packet of condoms because people would start thinking I am sleeping around so much, which will harm my reputation. I will get labelled. The same would apply to clinics also, when you go to get those free distributed ones.’ (P6, female, unemployed)

‘Some girls complain that public condoms cause rash and those from the shops have nice smell. I think that is the reason, smell and rash.’ (P3, male, college student)

Participants also reported the refusal of condom use by men because of the perception that they interfered with a sustained erection. Condoms were used at the initial stage during sexual intercourse but then dropped later because of the difficulty to sustain the erection. Some people preferred to use the withdrawal technique. This implies that there was more concern about preventing pregnancy than preventing sexually transmitted infections:

‘Some guys prefer to use condoms and remove them half-way. Their view is that their erection does not last when they use a condom. It may be a psychological problem or just an excuse. I had a couple of guys complaining about that and some other ladies reported the same to me.’ (P8, female, sales assistant)

There was an emerging belief that young adults were no longer afraid of HIV and AIDS because of the availability of antiretroviral drugs, freely provided in the public health sector, and the perception that treatment turned it into a manageable chronic disease:

‘As far as HIV is concerned, people used to be very scared back then when it was new, but nowadays given that people know that there is medicine and treatment, they do not really fear it as much. Treatment is also free. People now live longer with HIV.’

#### Opinion on the female condom

Some participants perceived that female condoms could be important in particular situations such as an abusive relationship, when the male partner is drunk or in case of prostitution. However, despite this acknowledgement, all female participants expressed their rejection of the female condom. The reasons being the size, having to keep them for hours in the vagina, the complicated insertion, as well as the fear of infection or other complications:

‘In situation where there is abuse, where the other partner is not considerate of the other, that is when the woman can take control. In this case, it can be very helpful. But I don’t know to what extent.’ (P2, female, nurse)

‘I have seen it, but never used it. And I do not think I will ever use it. I have never even tried it and do not ever want to. The reason being that it is too much work.’ (P2, female, nurse)

‘The way of inserting it is complicated. One would wonder where those rings will go and how they will settle on the inside and also if they are not going to cause any infection in the vagina.’ (P8, female, sales assistant)

#### Opinions on the HIV and AIDS prevention campaign

Most of the participants acknowledged and appreciated the effort and commitment of the government and non-governmental organisations in the fight against HIV and AIDS. They thought that the youth should take the opportunity offered to them and change their behaviours. However, some participants expressed their concerns about the current strategy. They suggested that effort should be made to better understand people’s attitudes and beliefs in their context and then to develop strategies to help them change their behaviours, rather than only promoting condom use. There was a need to take a more holistic and psychosocial approach to prevention rather than just a technical or biomedical one using interventions such as condoms. The safe male circumcision campaign might also mislead people to think that circumcision could provide total protection against the virus and make the use of condoms and safer sexual behaviour unnecessary:

‘The government is trying to help us as young people as the future of this country. I would say, for us as young people to appreciate what the government is trying to do to help us.’ (P4, female, university student)

‘They should focus more on changing the mindset, not the surgical things. They may work to some degree, but to me, I think there is still a long way to go because everything starts in the mind. They should come up with ways to change people’s mindset about condom use. Once they manage they will change the course.’ (P6, female, unemployed)

‘I believe the campaign will somewhat mislead people into believing that once circumcised, you can’t get infected.’ (P2, female, nurse)

#### Suggestions for a successful HIV and AIDS prevention campaign

The participants gave suggestions to improve the current preventive campaign against HIV and AIDS infection. They thought that the education on sexual issues and promotion of condom use should be incorporated in the curriculum from primary school. They also thought that this education should happen in the family. Parents should be encouraged to discuss these issues with their children. Families should be actively involved in the preventive campaign. They thought there was also a need to reach out to vulnerable people by means of a door-to-door campaign, which would also make it possible to engage with sexual partners when they might be together.

Some participants suggested putting in place strategies to change people’s behaviours. For them, without changing people’s mindsets about the use of condoms, the prevention campaign would never be effective. The quality of condoms distributed was also questioned by some participants. They thought that efforts should be made to improve their quality, for instance condoms should have a nice fragrance. For some participants, churches also played an important role in the campaign by educating people:

‘I think they have to teach those younger ones like those in primary school, very early about the disease and so on.’ (P10, male, technician)

‘Door-to-door campaign could be conducted. It will help and may be talking to partners when they are together can also help. If your partner cannot listen to you, at least the presence of a professional will help. Campaign at high-risk areas also will do some good.’ (P5, female, college student)

‘We do get the message from media. The problem is our parents. They can talk a little bit about it, but do not elaborate much, like to talk about the importance of what they are saying. It is just like a song.’ (P1, male, pharmacy assistant)

‘To eradicate or to reduce new HIV infections, the first thing to do is to target the mind. They should come up with ways to change people’s mindset.’ (P6, female, unemployed)

‘If something could be done about those [condoms] that are given free from the hospitals, maybe condoms would be used.’ (P6, female, unemployed)

‘The only way to prevent this is like joining a church. The will teach you a lot of things. Your mind will start to open up. But you are not forced.’ (P1, male, pharmacy assistant)

## Discussion

**FIGURE 1:**
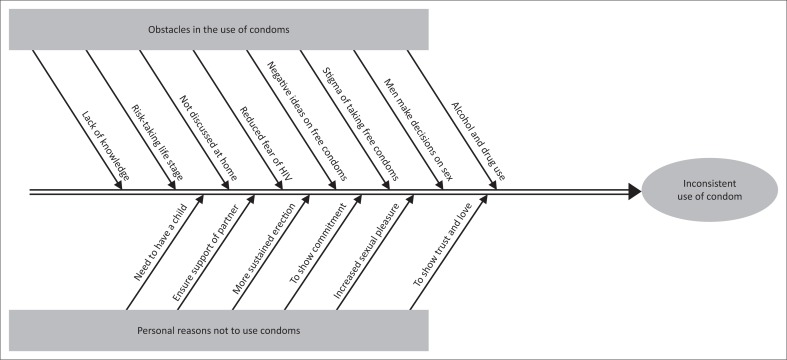
Fishbone diagram of barriers to using condoms by young adults in Mahalapye.

The principal obstacles and personal reasons to not use condoms among young adults in Mahalapye are summarised in Figure 1. Similar barriers to use of condoms were found in another study of Botswana youth: decreased pleasure, the influence of drugs and alcohol, trust of one’s partner, unavailability of condoms, influence of peers, unprotected sex as proof of love, desire for money and gifts.^23^ A study conducted in South Africa also identified the difficulties that families have in openly discussing issues of sex and HIV.^24^

The need to protect oneself from HIV and AIDS transmission by use of condoms may also be diminished by the belief that HIV is now a chronic disease that can be controlled better than other diseases such as cancer, diabetes or hypertension. In terms of the Heath Belief Model this could be interpreted as a loss of perceived severity leading to reduced motivation to protect oneself.^25^

The female condom was not accepted by the female participants. When women are supported in using the female condom and it is accessible, then their actual experience has led to acceptance and satisfaction.^24^ However, when women are asked for their opinion, without experience of the condom or support in using it, their viewpoint is often negative based on its unfamiliarity, unavailability or unaffordability.^24^ The female condom is considerably more expensive than the male condom, which also has economic implications for mass dissemination.^26^

Most participants appreciated the effort made by the government of Botswana in the current HIV and AIDS preventive campaign. Prevention has tended to focus on technological biomedical interventions such as using condoms or male circumcision and more recently antiretroviral medication. The need to also focus on socio-cultural issues and behavioural interventions was highlighted by the participants and has also been emphasised by the National Department of Health in South Africa.^27^ Behavioural interventions were found to be effective in promoting condom use and other safer sexual practices and reducing sexually transmitted infections.^28^

Some participants expressed their concern about the misleading message the male circumcision campaign might create among the youth. Another study conducted in Botswana, Namibia and Swaziland found that one in six men thought it was acceptable for a circumcised man to expect sex without a condom.^29^ Furthermore, in Uganda, a study indicated that the willingness to be circumcised was higher among those that had engaged in more risky sexual behaviours.^30^

### Limitations

Participants were selected from two facilities that were easily accessible to the researcher. It is possible that selecting youth from other facilities and communities in the area could have identified other themes. The researcher conducted all the interviews in English. An effort was made to include participants speaking only the local language; unfortunately none of them turned up for the interview. Including participants who were not able to speak English might have resulted in a wider variety of viewpoints and people with less education. Furthermore, interviews conducted by a male interviewer who was also the doctor could have had a negative influence on the openness of some participants.

### Recommendations

The following recommendations are based on the study findings:

- Preventive strategies should address the view that HIV and AIDS is a less serious threat to health now that treatment is available.
- Preventive strategies should focus on underlying socio-cultural issues and behaviour rather than just promoting technical solutions such as condoms and circumcisions.
- Improve the quality of freely distributed condoms (e.g. condoms with fragrance) and how they are displayed to allow more privacy when collecting them and reduce potential stigma.
- Parents and families should be enabled to discuss HIV and risky sexual behaviour with adolescents.
- The negative attitude towards female condoms could be addressed by interventions that make them accessible and support initial use.
- There is a need for more education for those undergoing male circumcision on the implications for safe sex and risk of transmission.

## Conclusion

This study explored the use of condoms in young sexually active adults in Mahalapye and identified a number of personal, relational and contextual reasons for inconsistent condom use. None of the women were keen to use the female condom. Participants were generally positive about the government’s HIV prevention campaign but thought there was an overemphasis on technical and biomedical solutions and more focus should be given to socio-cultural issues. They also expressed concern about misunderstandings regarding the effect of male circumcision on the risk of HIV transmission.
